# A Well‐Tolerated Hair Serum Containing New Natural Active Ingredients Reduced Hair Loss and Improved Quality of Life in Women With Chronic Telogen Effluvium: A 16‐Week Controlled Study

**DOI:** 10.1111/jocd.16656

**Published:** 2024-11-28

**Authors:** Virginie Turlier, Mélanie Froliger, Virginie Ribet, Valérie Mengeaud, Pascal Reygagne

**Affiliations:** ^1^ Département Développement et Expertise Clinique et Sensorielle R&D Pierre Fabre Dermo‐Cosmétique et Personal Care Toulouse France; ^2^ Direction Médicale, Laboratoires Dermatologiques Ducray Les Cauquillous, Lavaur France; ^3^ Centre de Santé Sabouraud Hôpital Saint‐Louis Paris France

**Keywords:** diffuse hair loss, quality of life, telogen effluvium, topical treatment

## Abstract

**Background:**

Chronic telogen effluvium (CTE) is characterized by hair loss lasting for more than 6 months and severely impairs quality of life (QoL). There is no specific treatment.

**Aims:**

To assess the dermatological tolerance and clinical efficacy of a hair serum containing three plant‐derived active ingredients (a *Silybum marianum* extract, Manganese PCA, and a *Lespedeza capitata* extract) in women with CTE.

**Methods:**

In this 16‐week, open‐label, two‐arm, controlled, parallel‐group study, 64 women aged 46 ± 10 years with CTE and a positive hair pull test were allocated equally to either a treated group (study serum and neutral shampoo) or a control group (neutral shampoo only).

**Results:**

The investigator rated dermatological tolerance of the serum as good at week (W)16. Compared to the control group, reductions in the amount of hair collected by the investigator during hair pull tests were greater in the treated group at W8 (*p* = 0.027) and W16 (*p* = 0.030), the amount of hair shed in 60 s (assessed by the subjects) was significantly lower in the treated group from W4, and more subjects in the treated group reported a decrease in hair loss over the study period (*p* ≤ 0.01; all time points). The treated subjects also perceived a significant improvement in hair volume, density, strength, and thickness, and reported being less depressed, less annoyed and less embarrassed by their hair loss.

**Conclusions:**

The serum was well‐tolerated and had a significant anti‐hair loss effect in women with CTE. This effect positively impacted QoL.

## 1 Introduction

Hair growth is a tightly regulated process characterized by cycles of three phases: growth (anagen phase), apoptosis‐mediated regression (catagen phase), and rest (telogen phase). These cycles follow a precise rhythm in healthy individuals, and cycle disturbances lead to hair loss and alopecia [1, 2, 3]. Telogen effluvium (TE) is a common type of alopecia in women and is characterized by non‐scaring, diffuse scalp hair loss, with an increase of telogen hairs and an increase in hair shedding of 7%–25% compared to normal [4]. Acute TE may be triggered by several factors including severe fever, chronic diseases, nutritional deficiency, endocrine imbalance, surgery and drugs [5, 6]. This acute hair loss lasts a few months before the hair grows back. Contrary to the acute form of TE, chronic telogen effluvium (CTE) lasts for more than 6 months [7, 8] and displays a fluctuating course over several years [5, 7]. Although CTE may be triggered by the same factors as the acute form, the chronic disorder is usually idiopathic, with no underlying cause identified in most cases [5, 6]. Healthy women are predominantly affected by CTE in their fourth to fifth decade of life [7].

Both scarring and non‐scarring forms of alopecia are known to have a psychosocial impact, which is more severe in women than in men, and to severely impair quality of life (QoL) [9, 10]. Indeed, anxiety, distress, depression, embarrassment, and decreased self‐esteem and confidence leading to social withdrawal, have been reported [9, 11]. CTE usually resolves spontaneously after 3–4 years, but may persist for over 10 years [12].

Despite the clear need for solutions to reduce the psychological burden of this hair loss disorder, there is no specific medical treatment for CTE [7]. Topical minoxidil 2% solution, approved for female AGA, can be useful in the management of CTE in women [6]. Although topical minoxidil is considered safe, associated side effects (e.g., irritant contact dermatitis on the scalp) are common. Moreover, unwanted hair growth in untreated areas has been reported, particularly in women receiving this treatment [13]. Alternative approaches, such as plant‐derived medicines and products, are currently being evaluated by both researchers and the cosmetics industry. In a previous study (Bacqueville et al., submitted as part of this supplement) three individual plant‐based active ingredients—a patented *Silybum marianum* extract (SME, WO/2021/023820), Manganese PCA (MnPCA), and a patented *Lespedeza capitata* extract (LCE, WO/2020/020791A1)—were found to display complementary anti‐hair loss properties in human follicle dermal papilla cells in vitro. In addition, a serum containing these three active ingredients was shown to stimulate hair matrix keratinocyte proliferation, promote the expression of factors involved in hair shaft anchorage, prolong the anagen phase and improve hair growth in a human ex vivo model. The aim of the current study was to assess the dermatological tolerance and the clinical efficacy of this hair serum in women with CTE.

## 2 Participants and Methods

### 2.1 Study Design and Setting

This open‐label, two‐arm, controlled, parallel‐group study was conducted by dermatologists in the Centre de Santé Sabouraud (Paris, France) between January and June 2021. Upon inclusion, all subjects were assigned a number: those with an odd inclusion number were allocated to the treated group and subjects with an even number were allocated to the control group. For each participant, the study lasted 16 weeks with five evaluation time points: Week 0 (W0, inclusion visit), W4, W8, W12, and W16. This study was performed in accordance with the Declaration of Helsinki and Good Clinical Practice Guidelines (EMA/CHMP/ICH/135/1995). In accordance with French regulations, as the study evaluated a cosmetic product, submission to an ethics committee was not required (Decree no. 2017‐884). All subjects provided written informed consent before enrolment, after having received verbal and written information about the study.

### 2.2 Participants

Women aged 18–65 years, with Skin phototypes I–V (Fitzpatrick classification) and a hair length ≥ 5 cm, and who had CTE for more than 6 months (diagnosed based on clinical and subject history and confirmed by trichoscopy), and a positive hair pull test [14] (≥ 10% of hairs pulled out from the scalp vertex) were eligible. Participants had to be using an effective method of contraception. Pregnant or breastfeeding women, and women who had frizzy hair, another hair disorder or hair disease (e.g., acute TE), an inflammatory skin disease, a progressive skin lesion on the scalp (psoriasis, seborrhoeic dermatitis, severe erythema, severe excoriation, severe sunburn, etc.), thyroid disorders stabilized for less than 3 months, history or clinical signs of hyperandrogenaemia (menstrual cycle > 35 days, hirsutism and signs of acne), or neoplastic disease at any time, as well as those with hypersensitivity to any of the serum components, those who were using another systemic or local anti‐hair loss treatment or product, or those who had undergone radiotherapy or chemotherapy at any time were considered ineligible. The use of concomitant treatments or interventions deemed likely to interfere with the study results was prohibited during the course of the study.

### 2.3 Products and Application Procedures

The test product (NEOPTIDE EXPERT Anti‐Hair Loss & Growth Serum, Ducray Laboratoires Dermatologiques, France) was a serum for the scalp and hair containing an SME (WO/2021/023820), MnPCA, and an LCE (WO/2020/020791A1) as active ingredients.

Subjects in the treated group were instructed to apply the serum to the scalp (damp or dry) every day, except after the last shampoo before a study visit, using a massaging motion with the fingertips to improve product penetration. The hair was not to be rinsed after application of the serum, and shampoos were prohibited within the 2 h after each application. Subjects were instructed to use a neutral shampoo (Ducray Extra‐gentle Shampoo) according to their usual routine. The first application of the serum was made at the study site, during the inclusion visit and under observation by the investigator. All other applications were performed at home under normal conditions of use. The subjects were asked to record each serum application in their daily log.

Subjects in the control group were instructed to use the neutral shampoo, according to their usual routine, except that they were asked not to wash their hair on the day of any of the scheduled study visits.

#### 2.3.1 Tolerance Evaluation

Tolerance was evaluated in subjects from both groups by assessing physical and functional dermatological signs at W0 (inclusion visit), before and during the 10–30 min after the first application of the serum for the treated subjects, and at W4, W8, W12, and W16. In the treated group, the investigators rated the individual tolerance for each subject, based on the presence or absence of any functional or physical signs (reactions likely or very likely) related to the test product, as excellent (no functional or physical signs), very good, good, moderate, or poor. The principal investigator then assessed global dermatological tolerance by attributing one of the same five ratings after taking into account the number and percentage of subjects with reactions related to the test product, the type of reactions, and their intensity, duration, time of emergence, and recurrence, as well as the number and percentage of subjects with similar reactions, the nature of test product, and the target population. Adverse events (AEs) were reported at each visit for all subjects.

#### 2.3.2 Efficacy Evaluation

The primary efficacy endpoint was the change between baseline (W0) and W4, W8, W12, and W16 in the amount of hair pulled out during scalp vertex hair pull tests performed by the investigators at each time point. Secondary efficacy endpoints included the change between W0 and W4, W8 and W12 in the number of hairs shed during 60‐s hair count tests performed by the subjects at home, and the change between W0 and W4, W8, W12, and W16 in subjective hair quality and in the subjects' hair loss assessment using a self‐administered questionnaire.

### 2.4 Methods

#### 2.4.1 Scalp Vertex Hair Pull Test

Approximately 50 hairs were grasped between the thumb and index fingers at the base of the hairs near the scalp and firmly, but not forcefully, tugged away from the scalp [14, 15]. The investigator then counted the number of hairs pulled out.

#### 2.4.2 60‐s Hair Count Test

The 60‐s hair count test was performed by the subjects at home according to a standardized method [16]. Briefly, the subjects combed their hair, using a comb provided by the study site, over the entire scalp for 60 s above a contrasting color surface, and then counted the number of hairs with a root that were either left on the comb or had fallen. The test was performed before each of the next three shampoos conducted after the study visits at W0, W4, W8, and W12.

#### 2.4.3 Subjective Evaluation of Hair Quality and Feelings About Hair Loss

Subjects completed a self‐assessment questionnaire to subjectively evaluate the quality of their hair (density, volume, strength, thickness, brittleness, dryness, and shine) and their feelings about hair loss (13 items regarding physical and psychological feelings) using visual analogue scales (Data S1).

### 2.5 Statistical Analysis

Analyses were performed on the full analysis set (FAS), which included all subjects who applied the test serum at least once. The results of the vertex hair pull tests were analyzed on both the FAS and the per protocol set (PPS: a subset of the FAS including all subjects with a primary efficacy criterion evaluation and without any major protocol deviations or other sources of bias for analyses of the primary criterion).

Intragroup comparisons (time effect) were performed using the paired *t*‐test, or the Wilcoxon test if the normality test was significant. Between‐group comparisons were performed using the unpaired *t*‐test if the assumption of normality of the distribution of the time effect for each group was not rejected, and the Mann–Whitney test if the normality test of the time effect was significant for at least one group.

Statistical analyses were performed using SPSS (v24.0). Statistical significance was set at 1% for the normality tests and at 5% for all comparisons.

## 3 Results

### 3.1 Participants

**FIGURE 1:**
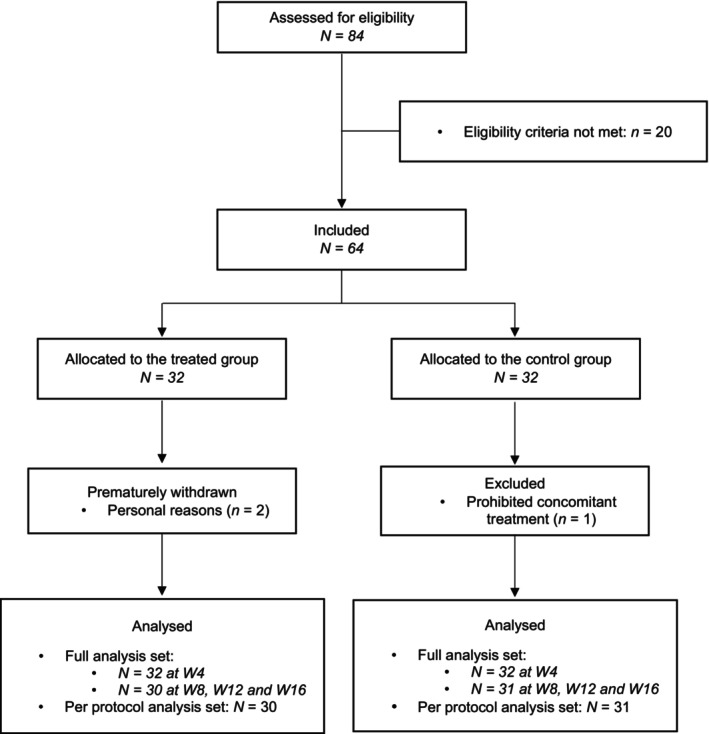
Flow chart of the study.

Overall, 64 women aged 46 ± 10 years with CTE were included: 32 in the treated group and 32 in the control group (Figure 1). No apparent between‐group differences in baseline demographic, hair, or clinical characteristics were observed (Table 1). Three subjects were excluded from the PPS: two subjects in the treated group withdrew at D58 for personal reasons, and one subject in the control group was excluded at D29 due to initiation of a prohibited concomitant treatment (iron supplements to treat anemia). Compliance was very good: only three subjects missed three (*N* = 2) or four (*N* = 1) serum applications over the 16‐week daily application period.

**TABLE 1:** Baseline characteristics of the subjects.

Parameters	Treated group (*N* = 32)	Control group (*N* = 32)	Overall (*N* = 64)
Age in years, mean ± SD (range)	47 ± 11 (25–63)	45 ± 9 (25–62)	46 ± 10 (25–63)
Skin phototype, *n* (%)			
II	3 (9.4)	3 (9.4)	6 (9.4)
III	25 (78.1)	24 (75.0)	49 (76.6)
IV	4 (12.5)	5 (15.6)	9 (14.1)
Menopausal stage, *n* (%)			
Non‐menopausal	20 (62.5)	23 (71.9)	43 (67.2)
Post‐menopausal	12 (37.5)	8 (25.0)	20 (31.2)
Pre‐menopausal	0 (0.0)	1 (3.1)	1 (1.6)
Scalp type, *n* (%)			
Dry	(18.8)	4 (12.5)	10 (15.6)
Normal	18 (56.2)	24 (75.0)	42 (65.6)
Oily	8 (25.0)	4 (12.5)	12 (18.8)
Scalp sensitivity, *n* (%)	4 (12.5)	5 (15.6)	9 (14.1)
Hair type, *n* (%)			
Dry	12 (37.5)	8 (25.0)	20 (31.2)
Normal	13 (40.6)	20 (62.5)	33 (51.6)
Oily	7 (21.9)	4 (12.5)	11 (17.2)
Hair length, *n* (%)			
Short	1 (3.1)	3 (9.4)	4 (6.2)
Middle	18 (56.3)	15 (46.9)	33 (51.6)
Long	13 (40.6)	14 (43.7)	27 (42.2)
Hair shape, *n* (%)			
Straight	9 (28.1)	7 (21.9)	16 (25.0)
Wavy	15 (46.9)	20 (62.5)	35 (54.7)
Curly	8 (25.0)	5 (15.6)	13 (20.3)

Abbreviations: *N* or *n*, number of subjects; SD, standard deviation.

### 3.2 Dermatological Tolerance and Safety

In the treated group, individual tolerance of the serum was rated as excellent by the investigators for 23/30 subjects (77%; Table 2) after 16 weeks of application. Among the subjects in the treated group that completed the study (*N* = 30), cutaneous and/or physical hair signs that were likely or very likely attributable to the test serum were observed in seven subjects (23%): scalp irritation reactions (*N* = 3, including one subject who had a recurrent episode of irritation that occurred prior to the study start), dry hair (*N* = 2), oily hair (*N* = 2), and matted hair (*N* = 1). The global dermatological tolerance of the test product, in association with the mild shampoo, was rated as good by the investigator after 16 weeks of application. In the control group, cutaneous and/or hair signs that were likely or very likely attributable to the shampoo were noted in 8/32 subjects (25%): mild or very mild scalp irritation was observed in four subjects, very mild diffuse sensitivity was reported by one subject, and dry, matted, or oily hair were observed in four subjects.

**TABLE 2:** Dermatological tolerance of the tested product.

	TOTAL (*N* = 30)
Individual tolerance	
Excellent^a^	23 (77%)
Very good	3 (10%)
Good	4 (13%)
Moderate	0 (0%)
Poor	0 (0%)
Reactions related to the tested product	
Total subjects with reactions related to the test product^b^, ^c^	7 (23%)
Functional and physical signs	0 (0%)
Functional signs only	0 (0%)
Physical signs only	7 (23%)
Subjects with reactions observed by the investigator	7 (23%)
Subjects with reactions likely or very likely attributable to the test product	7 (23%)
Subjects withdrawn from the study because of reactions to the test product	0 (0%)
Subjects who modified the modality of application and/or temporarily interrupted application of the test product because of a reaction	0 (0%)

^a^ Neither functional nor physical signs related to the tested product were observed by the investigator or reported by the subject.

^b^ Including both physical signs observed by the investigator and functional signs reported by the subjects.

^c^ Cutaneous functional signs (burning sensations, sensations of warmth, itching, skin tightness, stinging, or other) or cutaneous physical signs (erythema, oedema, desquamation, dry skin, vesicles, papules, seborrhoea, dandruff, or other) or hair physical signs (dry hair, hair loss, matted hair, oily hair, or other).

No AEs related to the test product occurred during the study period (Table S1). No serious AEs occurred.

### 3.3 Changes in Hair Loss From Baseline to W16: Pull Test (Primary Efficacy Criterion)

**FIGURE 2:**
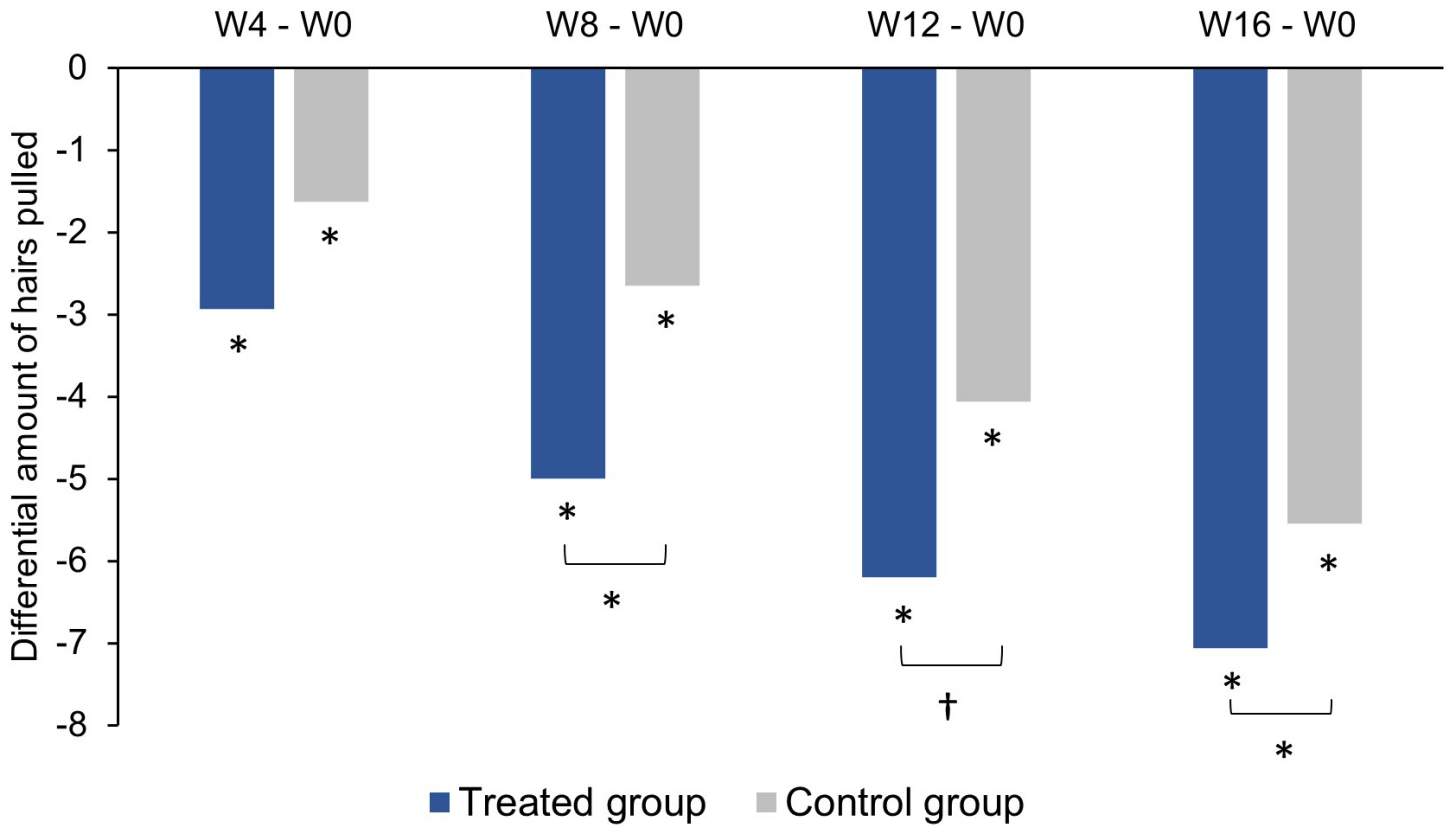
Changes in hair loss based on the results of hair pull tests performed on the scalp vertex by the investigator. Data are presented as the mean difference in the amount of hair pulled out (approximate percentage calculated as number of hairs pulled ×2) between each follow‐up time point (W4, W8, W12, and W16) and baseline (W0) for the full analysis set (FAS). *p < 0.05, ^†^ *p* = 0.0542 (W12). W, week.

The amount of hair collected from the vertex area was significantly lower in both groups at each follow‐up time point compared to baseline (Figure 2; Table S2). While significant reductions in the amount of hair pulled out were observed over time in both groups, the reduction was significantly greater in the treated group than in the control group at W8 (*p* = 0.027) and W16 (*p* = 0.030), and was close to significance at W12 (*p* = 0.054, Figure 2). There were no differences between the results of the FAS and the PPS analyses.

### 3.4 Changes in Hair Loss From Baseline to W16: 60‐s Hair Count Test

**FIGURE 3:**
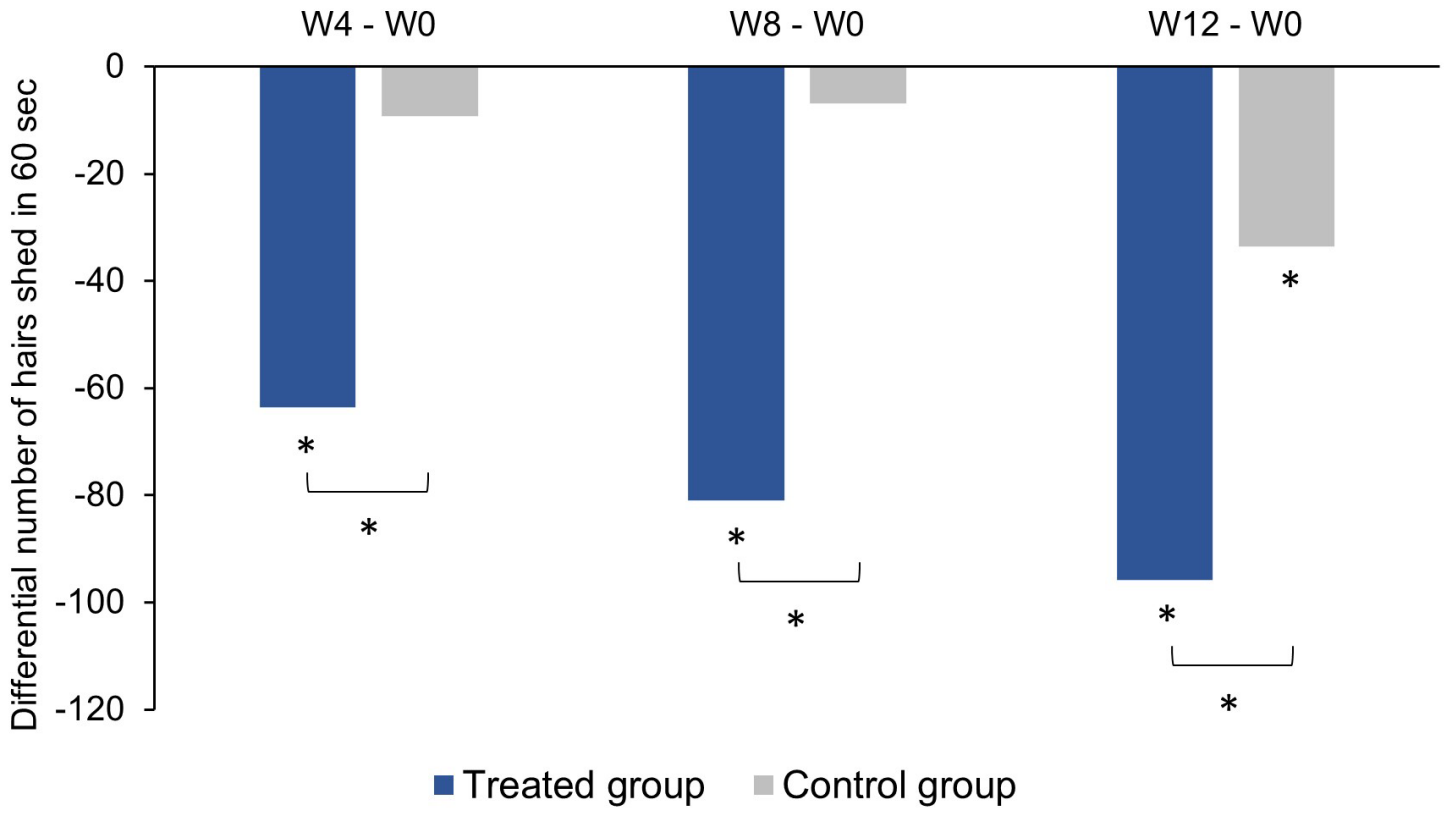
Changes in hair shedding based on the results of 60‐s hair count tests performed by the subjects at home. Data are presented as the mean difference in the total number of hairs shed between each follow‐up time point (W4, W8, and W12) and baseline (W0). *p < 0.05.W, week.

In comparison to baseline, the total number of hairs shed in 60 s was significantly lower at W4, W8, and W12 in the treated group, and at W12 only in the control group (Figure 3; Table S2). The size of the reduction in the total number of hairs shed in 60 s increased over time and was greater in the treated group than in the control group at each follow‐up time point (Figure 3).

### 3.5 Subjective Evaluation of Hair Quality

**FIGURE 4:**
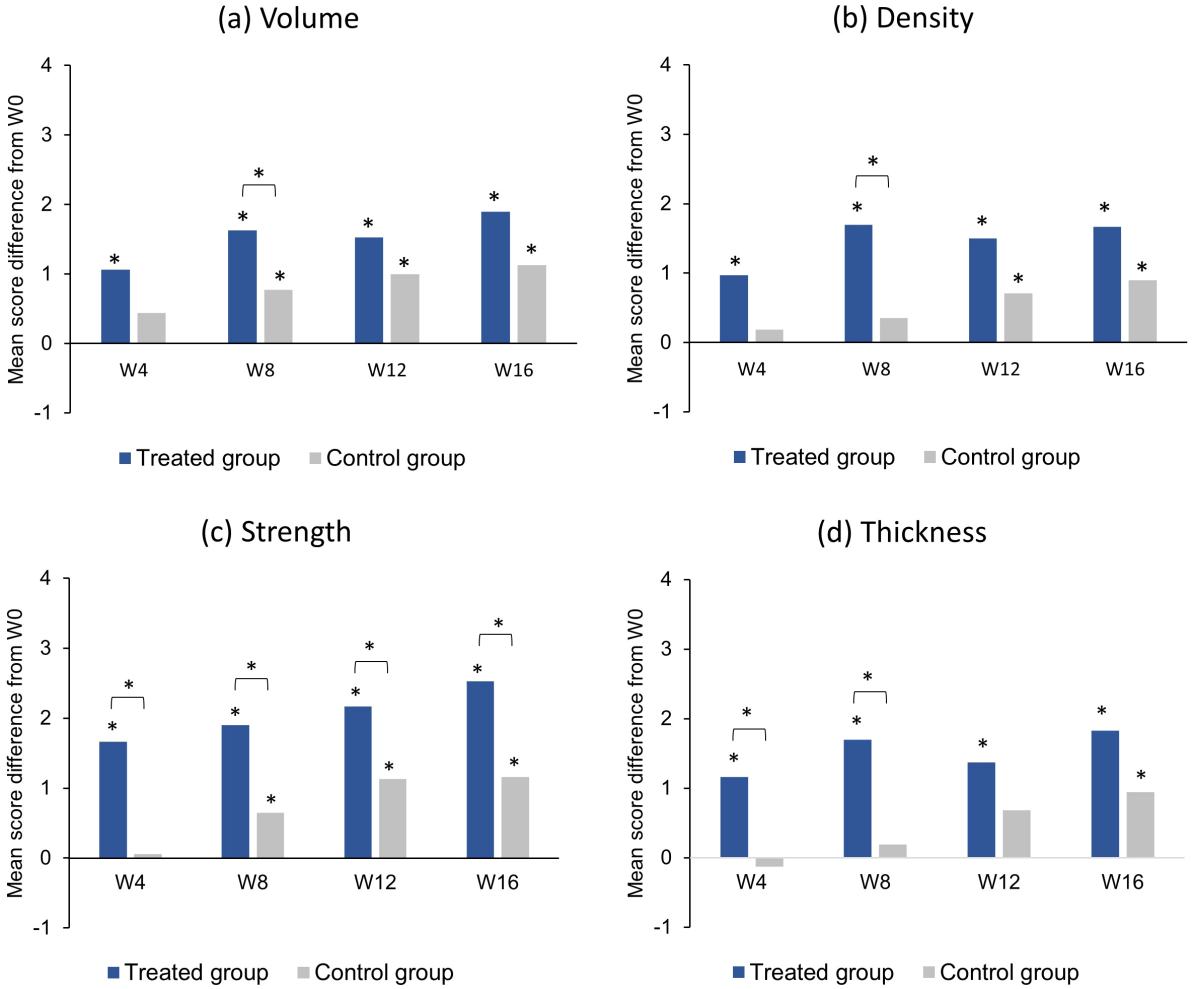
Evaluation of hair quality by the subjects based on responses to a self‐assessment questionnaire. Data are presented as the mean difference in scores, assigned on a 11‐point visual analogue scale (from 0 = “a very mild intensity” to 10 = “a very high intensity”), between each follow‐up time point (W4, W8, W12, and W16) and baseline (W0) for (a) hair volume, (b) hair density, (c) hair strength, and (d) hair thickness. *p < 0.05. W, week.

According to the self‐assessment questionnaire, women in the treated group perceived significant improvements from baseline in hair volume, density, strength, and thickness from W4 (Figure 4; Table S2). In comparison with the control group, a significant beneficial effect of the test serum was observed at W8 for hair volume and density, at W4 and W8 for hair thickness, and at each follow‐up time point from W4 to W16 for strength. No between‐group differences in brittleness, dryness, or shine were observed at any time point.

### 3.6 Subjects' Feelings About Their Hair Loss

**FIGURE 5:**
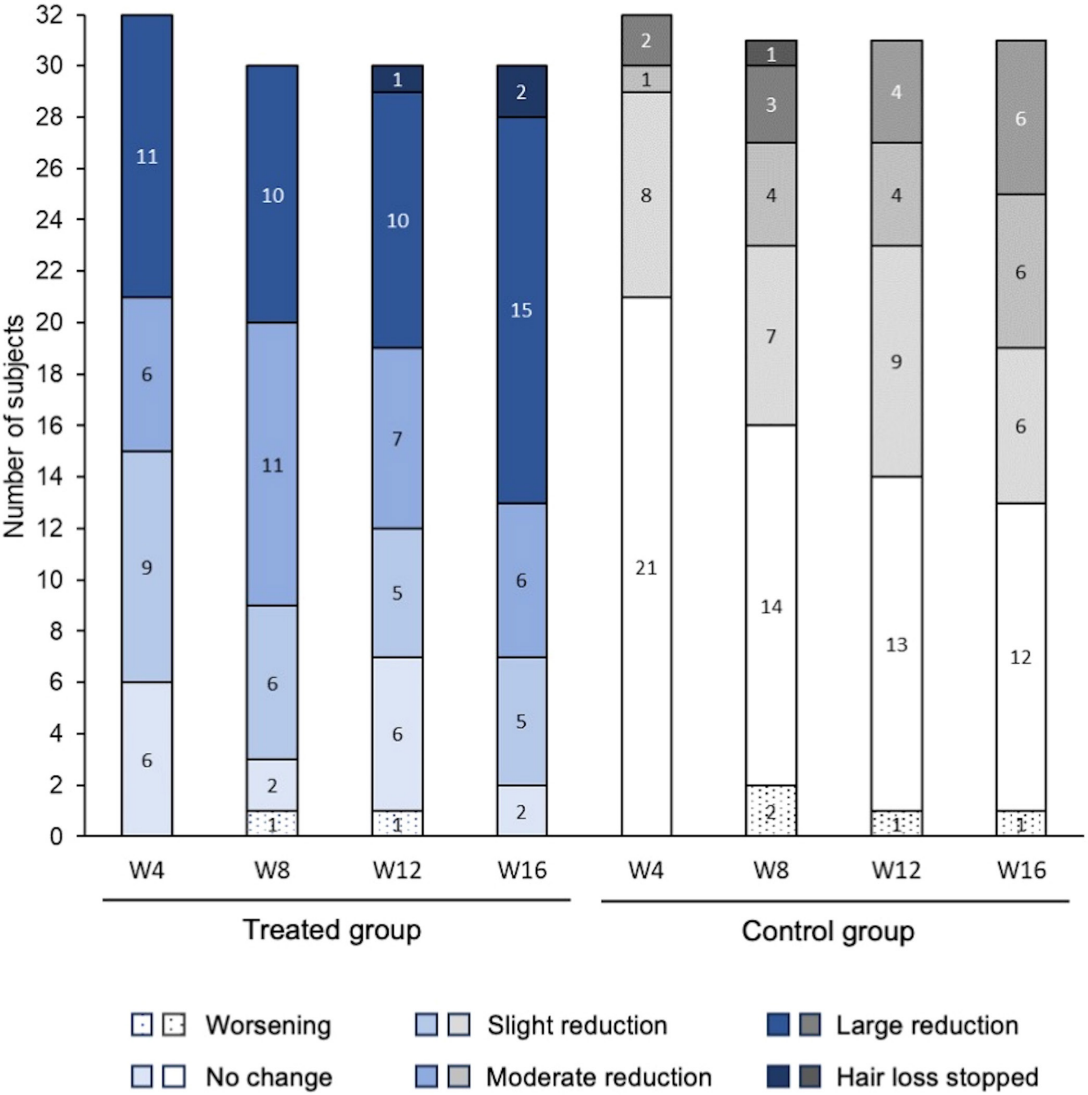
Evaluation of the change in hair loss by the subjects, based on responses to a self‐assessment questionnaire. Data are presented as the number of subjects reporting worsening of their hair loss, no change, a slight, moderate or large reduction in hair loss, or that their hair loss had stopped after 4, 8, 12, or 16 weeks of use of the test serum + neutral shampoo (treated group) or the neutral shampoo alone (control group). Three subjects (two subjects in the treated group and one subject in the control group) only answered this question at W4 due to their premature withdrawal or exclusion from the study. Between‐group differences: *p* < 0.01 at all‐time points. W, week.

**FIGURE 6:**
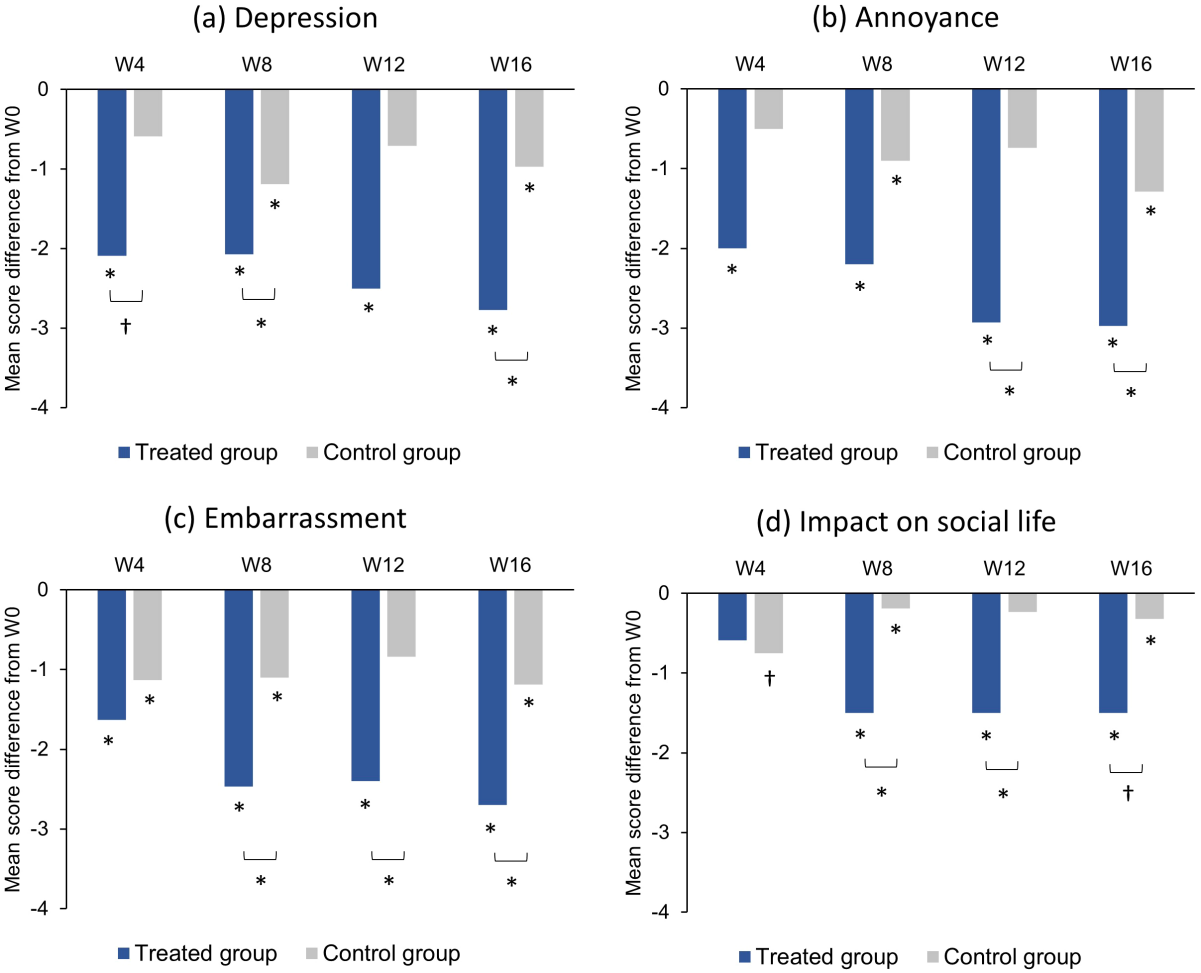
Evaluation of feelings about hair loss by the subjects based on responses to a self‐assessment questionnaire. Data are presented as the mean difference in scores, assigned on a 11‐point visual analogue scale (from 0 = “do not agree at all” to 10 = “totally agree”), between each follow‐up time point (W4, W8, W12, and W16) and baseline (W0) for (a) depression, (b) annoyance, (c) embarrassment, and (d) impact on social life linked to hair loss. *p < 0.05; ^†^ *p* = 0.0561. W, week.

The number of women reporting a decrease in hair loss was higher in the treated group than in the control group (Figure 5, *p* ≤ 0.01 at each time point). Moreover, in the treated group, decreases in scores for depression, annoyance and embarrassment with hair loss were observed from W4, and a reduction in the negative impact of hair loss on social life was reported from W8 (Figure 6; Table S2). Between‐group differences were significant from W8 for embarrassment, from W12 for depression and annoyance, and at W8 and W12 for impact on social life (Figure 6). No statistically significant between‐group differences were observed for the other evaluated items.

## 4 Discussion

In this controlled study, the newly developed hair serum containing three plant‐derived active ingredients was well tolerated and significantly reduced hair loss in women with CTE. Reductions in the number of hairs shed in 60 s and in the amount of hair collected during hair pull tests were observed in both groups over the course of the study; however, from W4 and W8 respectively, significantly greater reductions were observed for women who used the serum compared with those who did not. In the subjective assessments, a significantly higher proportion of women using the serum perceived a decrease in hair loss in comparison with women from the control group. The women who applied the serum also reported improvements in hair quality from W4 for most of the criteria evaluated, with significant between‐group differences observed for strength throughout the study, for thickness at the beginning of the study (W4 and W8), and for volume and density at W8 only. These improvements appeared to have a positive impact on QoL, with significant between‐group differences observed from W8 in favor of women using the study serum.

To date, no medical treatment has been approved for CTE. Topical minoxidil 2%–5% solution, which has been approved for other hair loss conditions such as AGA, can be prescribed off‐label for CTE. However, this treatment is effective in only a minority of women, and has often been associated with side effects such as irritation and allergic contact dermatitis, which manifest as scalp pruritus, scaling and itching, as well as with hypertrichosis and an increase in hair shedding during the first weeks of treatment [17]. Like other products containing natural compounds [18, 19], our study serum was well tolerated, with tolerance rated as excellent in 77% of the subjects and global dermatological tolerance rated as good. The good‐to‐excellent tolerability profile of the serum very likely contributed to the very high levels of compliance observed throughout the 16‐week study period.

Our results, obtained by using two non‐invasive and painless tests, showed that the study serum significantly reduced hair shedding from 4 weeks of use. These findings are consistent with those of our previous ex vivo study, which indicated that the combination of the three active ingredients contained in the test serum (SME + MnPCA + LCE) could be effective for reducing hair loss (Bacqueville et al., submitted as part of this supplement). Indeed, in this ex vivo study, topical application of the serum to human scalp skin samples enhanced hair shaft elongation, increased the proportion of hair follicles in the anagen phase, and stimulated the proliferation of hair matrix keratinocytes and the expression of keratin 75, which is involved in anchorage of the hair shaft [20, 21]. In vitro experiments performed in human follicle dermal papilla cells suggested that these effects could be explained by activation of the EGFR/PDGFR signaling pathways by SME, the modulation of the Wnt/β‐catenin pathway by MnPCA and LCE, and the stimulation of versican and vascular endothelial growth factor (VEGF) production by MnPCA (Bacqueville et al., submitted as part of this supplement). The Wnt/β‐catenin signaling pathway plays a key role in hair growth and the hair cycle, and the activation of this pathway by other plant‐derived products has been shown in vitro and in vivo in mice [18, 22, 23, 24, 25, 26, 27, 28].

To the best of our knowledge, very few, if any, controlled clinical studies have been conducted to evaluate the use of plant‐derived products in subjects with CTE. In a placebo‐controlled study [29], a combination of an herbal shampoo and a solution containing a mixture of six different herbal extracts was found to be effective in reducing hair loss in subjects with AGA and TE, probably through downregulation of interleukin‐1α [30]. However, it was not specified whether the subjects in this study presented with the chronic form of TE. The same goes for the placebo‐controlled trial involving ALRV5XR, an agent composed of compounds obtained from standardized botanical extracts, vitamins and minerals [31], and for the placebo‐controlled trial of a lotion containing a combination of zinc and arginine [32]. Both of these test products were found to have beneficial effects in women with AGA, TE, or hair loss occurring for more than 3 months, or in women and men with TE [32], but whether any of the subjects had CTE remains unclear. An extract from Edelweiss (*Leontopodium alpinum* var. Helvetia) has also been shown to have a beneficial effect in women and men with hair loss of unknown origin [25]. Finally, N1‐methylspermidine and resveratrol have been shown to be effective in counteracting hair loss in men and women with CTE when used in combination with Sandalore, a synthetic sandalwood‐like odorant that selectively stimulates olfactory receptors (OR)2AT4 [33].

We acknowledge several limitations in our study. Given the well‐established impact of seasonality on hair loss, it is important to consider seasonal factors in the evaluation of all hair loss studies [34]. Our study was performed between January and June, and thus included spring but not autumn (i.e., the two seasons during which seasonal hair loss is prone to occur). Our study was controlled, and the subjects were sequentially allocated to the treated group and the control group based on an even/odd number allocation sequence. Therefore, both groups would have been equally affected by any potential seasonal effects. Although not placebo‐controlled with an inactive serum, our study was controlled with the use of a neutral shampoo alone in a parallel control group. We recognize the potential bias related to the placebo effect, or to the effect of massaging the scalp for a very short time to facilitate product penetration. However, it is important to stress that the test serum contained very few non‐active ingredients included for galenic purpose, such as water, alcohol, PEG. There is so far no evidence showing an effect of such ingredients on the cellular mechanisms of hair loss. In addition, significant between‐group differences were observed for objective and subjective parameters, providing strong evidence supporting the effectiveness of the test serum, and allowing the possibility that the observed effects were due to spontaneous recovery to be excluded [8]. However, double‐blind placebo‐controlled studies are needed to confirm these findings. It would also be interesting to assess whether the effect of the study serum is maintained after the end of the application period. The women included in the current study presented with CTE. Assessing the efficacy of this serum in men and women with AGA would also be of interest, especially as two components of the test serum, LCE and MnPCA, have been shown respectively to reduce 5‐alpha reductase activity and to stimulate VEGF production in vitro.

In conclusion, the new serum containing an SME, MnPCA, and an LCE was well tolerated and had a significant anti‐hair loss effect in women with CTE. The reduction in hair loss was not only demonstrated quantitatively, but was also perceived by the women who applied the serum from 4 weeks of use, with most subjects reporting a significant reduction in their hair loss and an improvement in the quality of their hair, which also appeared to positively impact their QoL. This new serum could represent a good option for the management of women with CTE.

## Author Contributions

Protocol/project development: Virginie Turlier, Mélanie Froliger, and Valérie Mengeaud. Project administration and supervision: Virginie Ribet. Design, planning, conduct, and reporting of the study: Virginie Turlier, Mélanie Froliger, and Pascal Reygagne. Contribution to the design and implementation of the research: Valérie Mengeaud. Data collection, acquisition, and analysis: Pascal Reygagne. Interpretation of the results: Virginie Turlier, Mélanie Froliger, and Pascal Reygagne. Discussion of the results: Virginie Turlier, Mélanie Froliger, Valérie Mengeaud, and Pascal Reygagne. Critical review of the manuscript: Virginie Turlier, Valérie Mengeaud, and Pascal Reygagne. All authors reviewed the manuscript and approved the final version.

## Ethics Statement

The authors confirm that the ethical policies of the journal, as noted on the journal's author guidelines page, have been adhered to. This study was performed in accordance with the Declaration of Helsinki and Good Clinical Practice Guidelines (EMA/CHMP/ICH/135/1995). In accordance with French regulations, as the study evaluated a cosmetic product, submission to an ethics committee was not required (Decree no. 2017‐884). All subjects provided written informed consent before enrolment, after having received verbal and written information about the study.

## Conflicts of Interest

VT, MF, VR, and VM are employees or were employees of Pierre Fabre Dermo‐Cosmétique and Personal Care, France, at the time of the preparation of this manuscript and received salaries, but they do not have any financial interest in the findings described in this manuscript. PR received for his department honorarium fees for his role as investigator and for performance in this study.

## Supporting information


Data S1.

Table S1.

Table S2.

## Data Availability

The data that support the findings of this study are available on request from the corresponding author. The data are not publicly available due to privacy or ethical restrictions.
