# Supplementary material for: A Well‐Tolerated Hair Serum Containing New Natural Active Ingredients Reduced Hair Loss and Improved Quality of Life in Women With Chronic Telogen Effluvium: A 16‐Week Controlled Study
Source: J Cosmet Dermatol. 2024 Nov 28;23(Suppl 5):12–21. doi: 10.1111/jocd.16656 (PMC11603399; doi:10.1111/jocd.16656)
Supplement: Supplementary file 1 — Data S1. [file JOCD-23--s003.docx]

**SELF ASSESSMENT QUESTIONNAIRE**

**Hair quality evaluation**

***Please rate the intensity of each of the criteria below by circling a number between 0 and 10, with 0 corresponding to a very mild intensity and 10 corresponding to very high intensity.***

**Hair density**

| ⇦ **very mild Very High** ⇨ | | | | | | | | | | |
| --- | --- | --- | --- | --- | --- | --- | --- | --- | --- | --- |
| **0** | **1** | **2** | **3** | **4** | **5** | **6** | **7** | **8** | **9** | **10** |

**Hair volume**

| ⇦ **very mild Very High** ⇨ | | | | | | | | | | |
| --- | --- | --- | --- | --- | --- | --- | --- | --- | --- | --- |
| **0** | **1** | **2** | **3** | **4** | **5** | **6** | **7** | **8** | **9** | **10** |

**Hair strength**

| ⇦ **very mild Very High** ⇨ | | | | | | | | | | |
| --- | --- | --- | --- | --- | --- | --- | --- | --- | --- | --- |
| **0** | **1** | **2** | **3** | **4** | **5** | **6** | **7** | **8** | **9** | **10** |

**Hair thickness**

| ⇦ **very mild Very High** ⇨ | | | | | | | | | | |
| --- | --- | --- | --- | --- | --- | --- | --- | --- | --- | --- |
| **0** | **1** | **2** | **3** | **4** | **5** | **6** | **7** | **8** | **9** | **10** |

**Hair brittleness**

| ⇦ **very mild Very High** ⇨ | | | | | | | | | | |
| --- | --- | --- | --- | --- | --- | --- | --- | --- | --- | --- |
| **0** | **1** | **2** | **3** | **4** | **5** | **6** | **7** | **8** | **9** | **10** |

**Hair dryness**

| ⇦ **very mild Very High** ⇨ | | | | | | | | | | |
| --- | --- | --- | --- | --- | --- | --- | --- | --- | --- | --- |
| **0** | **1** | **2** | **3** | **4** | **5** | **6** | **7** | **8** | **9** | **10** |

**Hair shine**

| ⇦ **very mild Very High** ⇨ | | | | | | | | | | |
| --- | --- | --- | --- | --- | --- | --- | --- | --- | --- | --- |
| **0** | **1** | **2** | **3** | **4** | **5** | **6** | **7** | **8** | **9** | **10** |

**Hair loss evaluation**

***Please rate the extent to which you agree with each of the statements listed below concerning the condition of your scalp and your feelings about hair loss by circling a number between 0 and 10, with 10 signifying that you totally agree and 0 signifying that you do not agree at all.***

***Would you say that:***

|  | | ⇦ ***Do*** ***not agree at all Totally agree*** ⇨ | | | | | | | | | | |
| --- | --- | --- | --- | --- | --- | --- | --- | --- | --- | --- | --- | --- |
|  |  | 0 | 1 | 2 | 3 | 4 | 5 | 6 | 7 | 8 | 9 | 10 |
| 1 | ***Your scalp itches?*** | 0 | 1 | 2 | 3 | 4 | 5 | 6 | 7 | 8 | 9 | 10 |
| 2 | ***Your scalp is painful?*** | 0 | 1 | 2 | 3 | 4 | 5 | 6 | 7 | 8 | 9 | 10 |
| 3 | ***Your scalp is sensitive?*** | 0 | 1 | 2 | 3 | 4 | 5 | 6 | 7 | 8 | 9 | 10 |
| 4 | ***Your scalp is irritated?*** | 0 | 1 | 2 | 3 | 4 | 5 | 6 | 7 | 8 | 9 | 10 |
| 5 | ***You feel depressed about your hair loss?*** | 0 | 1 | 2 | 3 | 4 | 5 | 6 | 7 | 8 | 9 | 10 |
| 6 | ***You are annoyed by your hair loss?*** | 0 | 1 | 2 | 3 | 4 | 5 | 6 | 7 | 8 | 9 | 10 |
| 7 | ***You are embarrassed by your hair loss?*** | 0 | 1 | 2 | 3 | 4 | 5 | 6 | 7 | 8 | 9 | 10 |
| 8 | ***You are not satisfied with the condition of your hair?*** | 0 | 1 | 2 | 3 | 4 | 5 | 6 | 7 | 8 | 9 | 10 |
| 9 | ***You are stressed about hair loss?*** | 0 | 1 | 2 | 3 | 4 | 5 | 6 | 7 | 8 | 9 | 10 |
| 10 | ***Your hair loss affects your social life?*** | 0 | 1 | 2 | 3 | 4 | 5 | 6 | 7 | 8 | 9 | 10 |
| 11 | ***You are worried that your hair loss may be getting worse?*** | 0 | 1 | 2 | 3 | 4 | 5 | 6 | 7 | 8 | 9 | 10 |
| 12 | ***You are worried that hair loss may be incurable?*** | 0 | 1 | 2 | 3 | 4 | 5 | 6 | 7 | 8 | 9 | 10 |
| 13 | ***You have lost your self- confidence because of your hair loss?*** | 0 | 1 | 2 | 3 | 4 | 5 | 6 | 7 | 8 | 9 | 10 |

Additional question from week 4 to week 16:

***After X* weeks of use, did you notice any change in your hair loss?***

- - Yes
  - No

**If yes, is it:**

| a stop of the hair loss | 🞎 |
| --- | --- |
| a large reduction in the hair loss | 🞎 |
| a moderate reduction in the hair loss | 🞎 |
| a slight reduction in the hair loss | 🞎 |
| worsening of the hair loss | 🞎 |

*Note: Adapted for the assessment time points (4, 8 or 12 weeks). For week 16, the question began with “At the end of the 16 weeks”.
