# Supplementary material for: A Well‐Tolerated Hair Serum Containing New Natural Active Ingredients Reduced Hair Loss and Improved Quality of Life in Women With Chronic Telogen Effluvium: A 16‐Week Controlled Study
Source: J Cosmet Dermatol. 2024 Nov 28;23(Suppl 5):12–21. doi: 10.1111/jocd.16656 (PMC11603399; doi:10.1111/jocd.16656)
Supplement: Supplementary file 2 — Table S1. [file JOCD-23--s002.docx]

**Table S1. Adverse events reported during the study**

|  | **Treated group**  **N = 32** | **Control group**  **N = 32** | **TOTAL**  **N = 64** |
| --- | --- | --- | --- |
| **Number of subjects reporting adverse events** | 6 | 8 | 14 |
| **Number of adverse events** | 8 | 8 | 16 |
| **Number of serious adverse events** | 0 | 0 | 0 |
| **Number of severe adverse events** | 0 | 0 | 0 |
| **Number of adverse events leading to study withdrawal** | 0 | 1 | 1 |
| **Adverse events reported by ≥ 1 subject** |  |  |  |
| Anaemia* | 0 | 1 | 1 |
| Headache | 2 | 2 | 4 |
| Low back pain | 1 | 0 | 1 |
| Dorsalgia | 1 | 0 | 1 |
| Influenza-like illness syndrome | 1 | 0 | 1 |
| Covid-19 | 0 | 1 | 1 |
| Post-vaccination muscle arches | 0 | 1 | 1 |
| Allergic rhinitis due to pollen | 0 | 1 | 1 |
| Sinusitis | 1 | 0 | 1 |
| Otitis | 0 | 1 | 1 |
| Intestinal bloating | 1 | 0 | 1 |
| Spotting under the pill | 1 | 0 | 1 |
| Insomnia | 0 | 1 | 1 |

*The subject was excluded from the study because she took a prohibited concomitant treatment (iron supplement: Tardyferon B9)
