# Supplementary material for: A Well‐Tolerated Hair Serum Containing New Natural Active Ingredients Reduced Hair Loss and Improved Quality of Life in Women With Chronic Telogen Effluvium: A 16‐Week Controlled Study
Source: J Cosmet Dermatol. 2024 Nov 28;23(Suppl 5):12–21. doi: 10.1111/jocd.16656 (PMC11603399; doi:10.1111/jocd.16656)
Supplement: Supplementary file 3 — Table S2. [file JOCD-23--s001.docx]

**Table S2. Results of the quantitative and subjective efficacy evaluations over the course of the study**

|  | W0 | W4 | W8 | W12 | W16 |
| --- | --- | --- | --- | --- | --- |
| *Number of subjects* |  |  |  |  |  |
| *Treated group* | *32* | *32* | *30* | *30* | *30* |
| *Control group* | *32* | *32* | *31* | *31* | *31* |
| Hair pull test performed by the investigator | **W0** | **W4** | **W8** | **W12** | **W16** |
| Amount^a^ of hairs pulled out (mean, SD) |  |  |  |  |  |
| Treated group | 12.38 (2.35) | 9.44** (3.10) | 7.40** (2.69) | 6.20** (2.85) | 5.33** (2.12) |
| Control group | 13.25 (4.42) | 11.63** (3.39) | 10.65** (3.81) | 9.23** (3.45) | 7.74** (2.05) |
| 60-second hair count test performed by the subjects | **W0** | **W4** | **W8** | **W12** | - |
| Number of hairs shed in 60 seconds (mean, SD) |  |  |  |  |  |
| Treated group | 177.63 (159.39) | 121.37** (92.52) | 104.03* (64.10) | 89.17** (62.91) | - |
| Control group | 198.72 (159.95) | 165.68 (85.78) | 168.00 (82.02) | 141.29* (73.84) | - |
| Subjective evaluation of hair quality | **W0** | **W4** | **W8** | **W12** | **W16** |
| Volume (mean, SD) |  |  |  |  |  |
| Treated group | 4.31 (1.57) | 5.38* (1.98) | 6.07** (1.84) | 5.97** (1.99) | 6.33** (1.88) |
| Control group | 5.13 (2.71) | 5.56 (1.81) | 5.90* (1.62) | 6.13* (1.67) | 6.26* (1.61) |
| Density (mean, SD) |  |  |  |  |  |
| Treated group | 4.56 (1.93) | 5.53* (1.88) | 6.43** (1.77) | 6.23** (1.79) | 6.40** (1.54) |
| Control group | 5.41 (1.95) | 5.59 (1.60) | 5.81 (1.60) | 6.16* (1.70) | 6.35* (1.76) |
| Strength (mean, SD) |  |  |  |  |  |
| Treated group | 4.06 (1.61) | 5.72** (2.00) | 6.10** (1.97) | 6.37** (1.83) | 6.73** (1.96) |
| Control group | 4.69 (1.73) | 4.75 (1.81) | 5.35* (1.85) | 5.84* (1.51) | 5.87* (1.48) |
|  |  |  |  |  |  |
| Thickness (mean, SD) |  |  |  |  |  |
| Treated group | 4.31 (2.13) | 5.47* (1.97) | 6.20** (2.04) | 5.87* (1.89) | 6.33** (2.01) |
| Control group | 5.31 (2.51) | 5.19 (1.93) | 5.55 (1.73) | 6.03 (1.49) | 6.29* (1.66) |
| Feelings about hair loss | **W0** | **W4** | **W8** | **W12** | **W16** |
| Depression (mean, SD) |  |  |  |  |  |
| Treated group | 5.47 (3.17) | 3.38* (3.09) | 3.43* (3.20) | 3.00** (2.82) | 2.73** (2.80) |
| Control group | 3.47 (2.98) | 2.88 (2.78) | 2.35* (2.71) | 2.84 (2.83) | 2.58* (2.72) |
| Annoyance (mean, SD) |  |  |  |  |  |
| Treated group | 6.03 (2.82) | 4.03* (3.24) | 3.97* (3.25) | 3.23** (2.91) | 3.20** (2.83) |
| Control group | 3.84 (3.23) | 3.34 (2.80) | 3.00* (2.80) | 3.16 (2.93) | 2.61* (2.82) |
| Embarrassment (mean, SD) |  |  |  |  |  |
| Treated group | 5.94 (2.75) | 4.31* (3.29) | 3.53** (3.03) | 3.60** (2.85) | 3.30** (2.84) |
| Control group | 4.13 (2.96) | 3.00* (2.94) | 3.03* (2.66) | 3.29 (2.78) | 2.94* (2.49) |
| Impact on social life (mean, SD) |  |  |  |  |  |
| Treated group | 4.06 (3.11) | 3.47 (3.34) | 2.70* (3.15) | 2.70** (2.85) | 2.70* (2.78) |
| Control group | 2.31 (2.48) | 1.56 (2.06) | 2.03 (2.18) | 2.00 (2.38) | 1.90 (2.18) |

**Abbreviations:** SD, standard deviation; W, week. ^a^ Approximate percentage calculated as number of hairs pulled x2. * p‑value <0.05, ** p-value ≤0.0001 from baseline (W0)
